# Does Eosinophil Heterogeneity Translate into Functional Diversity? A Review of the Evolving Paradigm of Eosinophil Heterogeneity in Asthma

**DOI:** 10.3390/biomedicines12092011

**Published:** 2024-09-03

**Authors:** Gabriella E. Wilson, Samir Gautam, Geoffrey L. Chupp

**Affiliations:** Department of Internal Medicine, Yale School of Medicine, New Haven, CT 06510, USA; gabriella.wilson@yale.edu (G.E.W.); samir.gautam@yale.edu (S.G.)

**Keywords:** eosinophils, asthma, interleukin-5, inflammatory eosinophils

## Abstract

This review provides an overview of evidence supporting the existence of distinct homeostatic and inflammatory eosinophil subpopulations in health and disease. Particular emphasis is placed on describing the phenotypic and functional roles of these eosinophil subtypes in asthma, as well as the phenotypic changes induced by clinical therapy with the anti-IL-5 biologic agent, mepolizumab. Improved understanding of distinct eosinophil phenotypes may enable targeting of select subpopulations in the treatment of patients with type 2 inflammatory diseases such as asthma.

## 1. Introduction

Nearly 150 years after their discovery in 1879, the essential function of eosinophils remains a mystery, despite an ever-growing list of putative roles in health and disease [1]. Eosinophils originate in the bone marrow and get released into circulation in response to interleukin-5 (IL-5) [1]. They are recruited to tissues through a well-described sequence mediated by cytokines, chemokines, and adhesion molecules [2,3]. Historically, eosinophils have been implicated in the pathogenesis of helminthic infection and allergic diseases, but over the last decade evidence has emerged to suggest that they serve key functions under both homeostatic and inflammatory conditions [4]. More recent studies have proposed that the variable roles of eosinophils may be fulfilled by distinct cellular subpopulations. This framework may provide long-sought clarity in our understanding of eosinophils at the basal state and in disease and reveal mechanisms that may be targeted by novel therapies. Here, we provide a detailed review of eosinophil subtypes, focusing on how these advances have shifted our understanding of the eosinophil’s role in asthma pathogenesis.

## 2. Origins and Multiple Fates of Eosinophils

**Figure 1:**
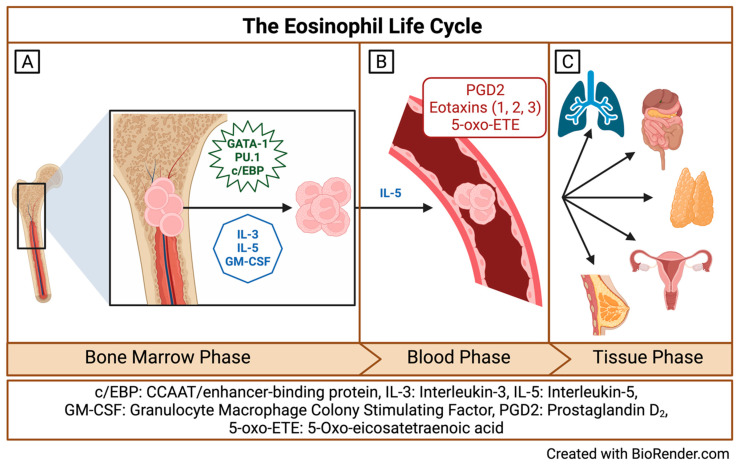
The Eosinophil life cycle. (**A**) The bone marrow phase of the eosinophil life cycle; (**B**) the blood phase of the eosinophil life cycle; and (**C**) the tissue phase of the eosinophil life cycle. Created with BioRender.com.

Eosinophils originate from CD34^+^ pluripotent progenitor cells in the bone marrow under the influence of several transcription factors including GATA-binding factor 1 (GATA-1), CCAAT-enhancer-binding protein (C/EBP), and PU.1 (Figure 1) [1,2]. Once committed to the eosinophil lineage, eosinophil progenitors express IL-5Rɑ, and eosinophil maturation occurs in the marrow under the influence of several cytokines including IL-5, interleukin-3 (IL-3), and granulocyte-macrophage colony-stimulating factor (GM-CSF). Eosinophils are released into the blood in response to IL-5 and under normal conditions comprise less than 5% of circulating leukocytes. The half-life of circulating eosinophils is 8–18 h, whereas that of eosinophils residing in tissues is estimated at 2–5 days [5]. Tissue residence may be even longer under certain conditions, such as after stimulation by IL-3, IL-5, GM-CSF, and IL-33 [5,6,7]. Within muscle and liver, eosinophils are major producers of interleukin-4 (IL-4), which is critical in promoting repair and regeneration in response to tissue injury [8,9]. Within the gastrointestinal (GI) tract, eosinophils produce APRIL, IL-6, and TGFβ, which function as plasma cell survival factors and are important for maintaining mucosal barrier integrity [10]. Eosinophils are also important in supporting metabolic homeostasis in adipose tissue by secreting IL-4 and, thus, favoring polarization of adipose macrophages toward an alternatively activated phenotype [11].

Eosinophils also play important roles in immune homeostasis in multiple compartments, e.g., by supporting B cell activation and plasma cell maturation in the bone marrow and by facilitating negative selection of auto-reactive T cells in the thymus [12,13]. In the setting of infection, eosinophils are equipped to fight a variety of pathogens through recognition by innate immune receptors, synthesis of pro-inflammatory cytokines, secretion of anti-microbial proteins, and production of extracellular mitochondrial DNA traps [1]. Studies conducted primarily in murine models suggest that eosinophils play a protective role in primary and secondary prevention of select helminthic infections [14], and recent work also suggests that eosinophils may be protective in the setting of viral, bacterial, and fungal infections [15]. 

Eosinophilopoiesis and eosinophil recruitment to the tissue is driven by type 2 inflammatory mechanisms in allergic diseases such as allergic rhinitis, asthma, and chronic rhinosinusitis [5,16]. In these conditions, various stimuli including allergens, microbes, and chemical irritants stimulate epithelial cells, mast cells, macrophages, and fibroblasts to secrete a variety of cytokines and alarmins including IL-25, IL-33, and thymic stromal lymphopoietin (TSLP). These factors stimulate Th2 cells to secrete IL-5, which drives eosinophil recruitment and activation. Additionally, IL-4 secretion from Th2 cells during the type 2 adaptive immune response also contributes to eosinophil recruitment [17]. Type 2 innate lymphoid cells (ILC2s) also secrete IL-5 in response to alarmins, thus enabling type 2 inflammation independently of adaptive immunity [18]. There is growing evidence that eosinophil migration into the tissue is also impacted by the microbiome in certain eosinophil-associated diseases. In particular, butyrate and propionate produced by commensal microorganisms have been shown to contribute to eosinophil survival and migration in asthma models through multiple mechanisms [19,20]. Following recruitment to the tissue during these innate and adaptive responses, eosinophils have the capacity to serve pro-inflammatory, anti-inflammatory, and regulatory roles [5]. However, the precise mechanisms by which eosinophils achieve this functional plasticity remain incompletely understood.

## 3. The Evolution of Eosinophil Subtypes in the Literature

The concept of eosinophil subtyping was first proposed in 1982 and 1983 following the identification of two distinct phenotypes based on density-gradient centrifugation, [21,22] similar to the pattern seen in neutrophils. This early work described “normodense” and “hypodense” eosinophil subsets in the peripheral blood of healthy volunteers and patients with eosinophilia and noted that hypodense eosinophils were more prominent in those with eosinophilia. They also proposed that “hypodense” eosinophils demonstrated a more activated phenotype based on decreased eosinophil cationic protein (ECP) content, increased oxygen consumption, increased expression of Fc-IgG and complement receptors, and higher capacity to induce cytotoxicity [21,22]. Increased numbers of hypodense eosinophils were later shown in the blood of patients with asthma and were found to correlate with clinical severity and airway hyper-responsiveness to methacholine [23,24]. 

In 2016, Abdala Valencia and colleagues employed a murine model of asthma to identify two compartment-specific eosinophil populations in the lung [25]. In this study, eosinophil recruitment was stimulated by an ovalbumin (OVA) challenge, and interstitial eosinophils from digested lung tissue were compared to airway eosinophils collected via bronchoalveolar lavage (BAL). Analysis of surface marker expression and functional adhesion assays demonstrated a transition from Siglec-F^med^CD11c^−^ cells in the interstitium to Siglec-F^high^CD11c^low^ eosinophils in the airway, indicating CD11c-mediated trans-epithelial migration during allergic inflammation. This work was also one of the first to highlight phenotypic diversity of eosinophils within a single organ. 

Following this pioneering work, Mesnil and colleagues undertook a detailed assessment of eosinophil subpopulations in a murine model of asthma [26]. This study identified two distinct eosinophil subsets in the lung, which they termed resident eosinophils (“rEos”) and inflammatory eosinophils (“iEos”). Resident eosinophils (“rEos”) were found within the lung parenchyma at a steady-state and exhibited a ring-shaped nucleus with secondary granules demonstrating variable levels of a density suggestive of piecemeal degranulation, a process whereby eosinophils selectively package granule proteins into vesicles for release at the cell surface via vesicle fusion with the cell membrane. An allergen challenge with house dust might (HDM) did not change the phenotype or parenchymal localization of rEos compared to the steady-state. However, after the challenge, an additional subpopulation of eosinophils (iEos) was identified in the peribronchial interstitium and bronchoalveolar space. iEos were structurally and phenotypically distinct from rEos, with a highly segmented nucleus with dense secondary granules without signs of degranulation. Flow cytometric analyses identified L-selectin (CD62L) as a specific marker for rEos across conditions (steady-state vs. HDM-treated) and showed that CD62L was consistently absent on iEos. Meanwhile, CD101 was noted to be highly expressed on iEos and nearly absent on rEos. CD62L was also assessed in eosinophils isolated from the small intestine, given the large reservoir of tissue-resident eosinophils known to reside there. Interestingly, these steady-state intestinal eosinophils did not express CD62L, suggesting that CD62L expression may be specific to rEos isolated from the lung and indicating that tissue-resident eosinophils exhibit phenotypic diversity depending on the organ site.

Mesnil and colleagues also performed functional and transcriptomic analyses to characterize the eosinophil subsets [26]. Despite similar expression of IL-5Rɑ, in vivo studies with anti-IL-5 treatment suggested that the presence of rEos in the lung is IL-5-independent. Transcriptomic profiles of rEos were similar in the steady-state and after an allergic challenge. Conversely, iEos exhibited a distinct transcriptomic signature after a challenge, with over 160 differentially expressed genes compared to rEos. Specifically, iEos demonstrated high expression of pro-inflammatory genes such as *IL13Ralpha1*, *Il6*, *Tlr4*, *Slc3a2*, and *C3ar1*. In contrast, rEos exhibited high expression of several genes involved in the suppression of type 2 inflammation, including *Runx3*, *Anxa1*, and *Ldlr*. The latter finding was further supported by in vivo experiments comparing characteristics of mediastinal lymph node (MLN) cell proliferation and cytokine production between rEos-deficient mice (ΔdblGATA) and control mice following an HDM-challenge. These in vivo experiments demonstrated increased proliferation and Th2 cytokine production (IL-4, IL-5, and IL-13) within MLN cells from HDM-treated ΔdblGATA mice compared to HDM-treated control mice. Additionally, α–IL-5- treated iEos-depleted WT mice did not exhibit increased susceptibility to HDM-induced Th2 responses compared to control mice, indicating that rEos are responsible for mediating Th2 cytokine production in response to HDM [26].

Finally, the Mesnil group used translational models to evaluate the rEos/iEos paradigm in humans [26]. Lung tissue from five healthy donors and five patients with asthma was analyzed using Congo red and staining for major basic protein (MBP), a general eosinophil marker; this revealed similar numbers of parenchymal eosinophils in both groups (presumed to represent rEos). Additional eosinophils were noted in the peribronchial areas among the subjects with asthma (presumed to represent iEos). They also isolated eosinophils from normal lung tissue (presumed rEos) and sputum of asthmatic subjects (presumed iEos). No differences in morphology were found between the two eosinophil populations, but iEos were consistently negative for CD62L, whereas rEos were noted to express variable levels of CD62L, as in the mouse model. Additionally, they found that IL-3R was significantly higher in iEos compared to rEos, leading to the suggestion that CD62L and IL-3R may be useful markers to distinguish rEos and iEos in humans, respectively. This was the first study to convincingly demonstrate that distinct eosinophil subpopulations exist in the lungs, and it has served as the foundation for several eosinophil subtyping studies since, as described below.

Andreev et al. later evaluated the concept of discrete eosinophil subpopulations in a murine model of resolving rheumatoid arthritis (RA) [27]. In this model, mice underwent intraperitoneal injection of pooled serum from arthritic adult mice to induce arthritis, and then asthma was elicited using OVA. Interestingly, the OVA challenge led to an accumulation of eosinophils in the synovium and was associated with rapid resolution of arthritis along with preservation of the joint structure. They termed this subset of synovial cells “regulatory eosinophils” and found that it expanded in response to IL-5, akin to the IL-5 dependent, recruitable “iEos” identified in the lung by Mesnil et al. Furthermore, IL-5 blockade, in this model, blocked asthma-induced resolution of arthritis and led to the attenuation of eosinophil accumulation in the lung (iEos). Single-cell RNA-sequencing analyses of eosinophils isolated from the lung and synovium of OVA-challenged mice confirmed that the rEos in the synovium were distinct from iEos isolated from the lung tissue. Importantly, this group also reported on eight patients with RA in remission and eosinophilic asthma who were treated with IL-5 blockade using mepolizumab. Of these eight patients, six experienced a flare of their RA after the initiation of mepolizumab therapy. These findings support the presence of distinct eosinophil subpopulations in different tissue contexts and indicate that eosinophils within the synovium may serve immunomodulatory and regenerative functions. Additionally, these findings imply that eosinophils from distinct compartments may be impacted differentially by anti-IL-5 treatment due to variable dependence on IL-5.

Considering these findings and the known heterogeneity of neutrophils and macrophages in response to type 1 stimuli (e.g., IFNɣ and bacteria) vs type 2 stimuli (e.g., IL-4 and IL-13) [28,29], Dolitzky et al. undertook studies to examine the transcriptional spectrum of eosinophils following type 1 vs type 2 stimulation [30]. Specifically, eosinophils were isolated from the peritoneal cavity of IL-5 over-expressing mice (C57BL/6 NJ.1638 *Il5^Tg^*) and treated in vitro with heat-inactivated *Escherichia coli* +/− IFNɣ, or IL-4. RNA sequencing demonstrated a distinct transcriptome signature among eosinophils following type 2 stimulation with IL-4, including the upregulation of *CD101* (a marker previously shown to be specific to iEos [26]) and *CD69* (a marker shown to be expressed by activated eosinophils [31]). Additionally, IL-4 stimulation increased the expression of several soluble mediators including *Ccl22*, *Ccl24*, *Ccl8*, and *Ccl12* and multiple transcription factors that are known to be upregulated in alternatively activated macrophages, such as *Pparg* and *Irf4*. Eosinophils stimulated with *E. coli* exhibited increased expression of pro-inflammatory cytokines including *IL6*, *IL1b*, and *TNFa*; type 2 cytokines *IL13* and *IL9*; and several genes involved in innate immune responses. Stimulation with IFNɣ induced a transcription signature consisting of upregulated cell surface receptors *CD274* (programmed death ligand 1), *Ly6a*, *Il13ra1*, and leukocyte-associated immunoglobulin-like receptor 1 (*Lair1*), as previously shown [32], as well as cell surface markers present on classically-activated macrophages, including *CD86*, *CD53*, and *CD36* [33]. Various secreted factors were upregulated following IFNɣ stimulation as well, including *CXCL9*, and downregulation was noted in markers related to allergy and asthma, including *CD48, Tgfbi*, and *Car4* [34,35]. Transcriptomic analysis following stimulation with both IFNɣ, and *E. coli* demonstrated substantial overlap between the transcriptome signatures following type 1 activation with IFNɣ. The authors then compared the transcriptome of eosinophils isolated from the colon of mice undergoing dextran sulfate sodium (DSS)-induced colitis (a model characterized by high exposure to microbes and IFNɣ) and eosinophils isolated from the lungs of mice with experimental asthma (characterized by high exposure to IL-4) to the transcriptomes of type 1- and type 2-stimulated eosinophils from their in vitro experiments. They found that the transcriptome of eosinophils from the DSS-induced colitis model clustered with the type 1-stimulated eosinophils, and the transcriptome from the asthma model clustered with the type 2-activated eosinophils. This study provides evidence for unique transcriptional profiles among eosinophils following different stimuli and further extends our understanding of eosinophil plasticity. Furthermore, this study identified potential markers that may be useful to distinguish type 1 from type 2-activated eosinophils including CD274 and CD53 for type 1 activation and CD101 and CD34 for type 2 activation.

Using a similar approach, Gurtner et al. analyzed the transcriptomic signatures of eosinophils isolated from various organs of IL-5-overexpressing mice [36]. Clustering analysis revealed five eosinophil subpopulations at different stages of development with immature eosinophils primarily present in the bone marrow, circulating eosinophils in the blood, and two subsets in the GI tissue, which were termed active eosinophils (A-Eos) and basal eosinophils (B-Eos). Relative proportions of A-Eos and B-Eos were tissue-specific within the GI tract, with B-Eos predominating in the stomach, A-Eos predominating in the colon, and an equal balance in the small intestine. B-Eos were noted to express effector molecules with roles in tissue morphogenesis and remodeling, including *Mmp9* and *Tgfb1*, whereas A-Eos were noted to express co-stimulatory molecules *CD80* and *CD274*, suggesting a role in immune modulation. A-Eos were also noted to express soluble inflammatory factors (*Il16*, *Tnfa*, *Il1b*, *Ccl3*, *Cxcl2*, *Vegfa*, and *Ptgs2*) and receptors (*Tgfbr2*, *Ccr2*, and *Cxcr4*). Proteome analysis from eosinophils isolated from blood, small intestine, and colon of wild-type mice revealed that A-Eos could be identified by PD-L1 and CD80 expression, and were noted to exhibit higher secretory activity, granularity, and activation relative to B-Eos. A-Eos were also noted to localize closer to the intestinal epithelium compared to B-Eos, which localized to the submucosa. This study also identified A-Eos and B-Eos in the GI tract of patients with inflammatory bowel disease (IBD) and healthy controls using immunofluorescence assays to MBP and PD-L1 and found that A-Eos (MBP^+^PD^−^L1^+^) were localized closer to the lumen than B-Eos (MBP^+^PD^−^L1^−^) and were substantially increased in patients with IBD compared to healthy controls. 

Gurtner et al. then examined these eosinophil subpopulations using three models of GI inflammation to better understand their functional roles [36]. In all three models, acute *Citrobacter rodentium* infection in the colon, chronic *Helicobacter pylori* infection in the stomach, and DSS-induced colitis, A-Eos were increased compared to B-Eos and demonstrated upregulation of genes involved in granulogenesis, antimicrobial activity, immune modulation, IFNɣ signaling, and major histocompatibility complex class I (MHC-1)-restricted antigen processing and presentation. Furthermore, single-cell fate probability computations, trajectory inference, and RNA velocity analysis revealed A-Eos to be the predicted terminal state for all eosinophil subsets both at steady-state and during infection and indicated that A-Eos originate directly from immature eosinophils during infection but arise from B-Eos in the steady-state. In vitro analyses from this study revealed that IL-33 plays an important role in inducing maturation of bone marrow-derived eosinophils to A-Eos. This study further supports the notion that two distinct eosinophil subpopulations are present in the GI tract and was the first to propose that these subpopulations exist on a differentiation continuum.

## 4. The Significance of Eosinophil Subpopulations in Asthma

Several other groups have sought to characterize eosinophil subpopulations in patients with asthma, primarily using the framework of CD62L^hi^ rEos and CD62L^lo^ iEos outlined by Mesnil et al. Januskevicius et al. was the first to identify rEos and iEos in the blood of patients with asthma separated by phenotype [37]. In this study, blood eosinophils were isolated from severe non-eosinophilic asthmatic patients at a single time point, and from allergic asthmatic patients and healthy subjects at two time points before and after a bronchial challenge with *D. pteronyssinus* allergen. Eosinophils were enriched from isolated granulocytes using magnetic-activated cell sorting (MACS), with CD62L+ eosinophils labeled as rEos and unlabeled cells identified as iEos. Adhesive properties and viability of rEos and iEos were then assessed after co-culture with healthy airway smooth muscle (ASM) cells. The authors found that iEos predominated in the blood of allergic asthmatic patients at baseline, whereas rEos were the predominant eosinophil subtype present in the blood of patients with severe non-allergic eosinophilic asthma, and healthy subjects exhibited roughly equal proportions of rEos and iEos. Furthermore, the relative predominance of iEos in the blood of allergic asthmatics was reversed following bronchial challenge. These findings indicate the presence of rEos and iEos in the blood, and the attenuation of iEos following the allergen challenge supports the notion that iEos are a recruitable subset of eosinophils that migrate into the tissue in response to an allergen challenge. Furthermore, they found that rEos exhibited increased adhesive properties compared to iEos in all study groups, supporting the idea that this subset of eosinophils exists as resident cells within tissue. A bronchial challenge in patients with allergic asthma led to increased viability and adhesion of both rEos and iEos.

Eosinophils have also been shown to contribute to ASM remodeling directly through integrin-mediated binding to ASM cells in response to T2 chemokines [38,39]. In a separate study, Jurkeviciute et al. evaluated integrin expression by eosinophil subtype, as well as the effects of eosinophil subtypes on ASM cell proliferation and viability [40]. They similarly examined patients with allergic asthma, severe non-allergic eosinophilic asthma, and healthy controls and utilized similar methods to separate rEos from iEos in the blood. They found that rEos exhibited higher transcriptional expression of β1 integrin compared to iEos, which exhibited higher expression of the ɑMβ2 integrin subunits. These changes in expression may underlie functional differences between rEos and iEos, though further studies are needed to confirm this. Assessment of the effects of eosinophil subtypes on the proliferation of ASM showed that both significantly increase ASM proliferation, indicating a conserved effect of eosinophils on airway remodeling. 

More recently, Cabrera Lopez et al. examined eosinophil subpopulations in patients with asthma, chronic obstructive pulmonary disease (COPD), smokers without COPD, and healthy volunteers [41]. In this study, eosinophil subpopulations were identified in the blood by flow cytometry using the “Mesnil criteria”, which labeled rEos as Siglec-8+, CD62L+, IL3R^lo^ and iEos as Siglec-8+ CD62L^lo^ IL3R^hi^. The authors found that circulating iEos were significantly higher in patients with asthma compared to the other groups and that the proportion of iEos remained higher in patients with asthma compared to COPD when matched by eosinophil count. They also found that iEos from the asthmatic group exhibited significantly higher expression of IL-5Rɑ compared to healthy subjects, smokers without COPD, and patients with COPD [41]. 

Matucci and colleagues have performed two detailed studies examining eosinophil subtypes in patients with asthma [42,43]. In these studies, granulocytes were isolated from peripheral blood, and fluorescence-activated cell sorting (FACS) was used to separate eosinophil subpopulations based on expression of Siglec-8 and CD62L (i.e., CD62L^bright^ rEos and CD62L^low^ iEos). In the first report, iEos and rEos were identified in blood and nasal polyp tissue from patients with severe eosinophilic asthma with and without chronic rhinosinusitis with nasal polyposis [42]. Once again, this study found that iEos were increased in the blood of patients with severe eosinophilic asthma compared to healthy donors and that iEos were highly concentrated in nasal polyp tissue from patients who had concomitant chronic rhinosinusitis with nasal polyposis [42,43]. The authors also examined surface marker expression among the two eosinophil subpopulations and found that blood iEos demonstrated increased the expression of CCR3 and CD69 as compared to rEos, supporting the notion that iEos reflect an activated subset of eosinophils that are likely functionally distinct from rEos [31]. They also demonstrated that iEos expressed lower levels of IL-5Rɑ, CRTH2, CD86, and CD28 compared to the rEos. 

More recently, Vultaggio et al. examined blood eosinophil subtypes in patients with severe eosinophilic asthma with and without chronic rhinosinusitis with nasal polyposis before and after mepolizumab treatment [43]. This study found a weak but statistically significant correlation between the proportion of CD62L^low^ eosinophils (“iEos”, percent of total isolated Siglec8^+^CD16^−^ eosinophils) and asthma control parameters, as assessed by the Asthma Control Test (ACT), the Asthma Control Questionnaire-5 (ACQ-5), and history of asthma exacerbations [43]. This finding suggests that iEos may have a role as a biomarker for risk of exacerbations and poor asthma control. They also isolated eosinophils from the blood for culture and examined changes in eosinophil subpopulations following stimulation with several different cytokines and alarmins. Stimulation with IL-5 resulted in a significant increase in the percentage of CD62L^low^ eosinophils, and exposure to IL-5 blockade with mepolizumab and an anti-IL-5R antibody abrogated this expansion of CD62L^low^ eosinophils. In vivo analyses of patients with severe eosinophilic asthma revealed that treatment with mepolizumab significantly reduced the proportion of CD62L^low^ eosinophils in the blood (out of all eosinophils isolated). Furthermore, the reduction in CD62L^low^ eosinophils induced with mepolizumab treatment during this study was shown to be sustained over time and was noted to directly correlate with improvement in post-treatment ACT and ACQ-5 scores. These novel findings suggest that mepolizumab may improve asthma control through a direct effect on reducing circulating iEos, effectively restoring a “healthy balance” among eosinophil subtypes in patients with severe eosinophilic asthma.

Our work has focused on characterizing eosinophil subtypes in the sputum from children with asthma and examining the relationship between eosinophil subtypes and asthma exacerbations. Mepolizumab for urban children with exacerbation-prone eosinophilic asthma in the USA (MUPPITS-2) was a multi-center, randomized, placebo-controlled clinical trial to determine the efficacy of mepolizumab in reducing asthma exacerbations among high-risk urban children with eosinophilic asthma [44]. This study demonstrated that mepolizumab reduces asthma exacerbations by 27% compared to placebo and significantly reduces blood and nasal eosinophils. In a sub-study to this trial, our group sought to identify eosinophil subpopulations in the airways of children with asthma using mass cytometry (CyTOF) [45]. Sputum was collected from 53 participants at four of the MUPPITS-2 sites and was analyzed using CyTOF. Unsupervised clustering of manually gated sputum eosinophils revealed three distinct subpopulations with low, intermediate, and high expression of CD62L (“CD62L^lo^”, “CD62L^int^”, and “CD62L^hi^” eosinophils). This variability of CD62L expression is distinct from that seen in previous studies, which may relate to the compartment sampled (sputum) or the sensitivity of detection with CyTOF. Furthermore, in our study, CD62L^int^ eosinophils exhibited significantly elevated expression of several chemokine receptors and ligands involved in eosinophil activation and mobilization, including CD69, CD80, ICAM1, MIP1β, Eotaxin, and CCR3. Similar to iEos from the Mesnil et al. study, CD62L^int^ eosinophils also exhibited significantly increased expression of IL3R compared to the other eosinophil subpopulations. Among mepolizumab-treated participants, CD62L^int^ and CD62L^hi^ eosinophils were more abundant in participants who experienced exacerbations during the study than in those who did not. Notably, the number of CD62L^int^ eosinophils was not higher in patients in the placebo group who had exacerbations. Taken together, our results suggest that sputum eosinophils with intermediate CD62L expression exhibit an activated phenotype and may correspond to the iEos identified in the blood from prior studies. Additionally, our study uniquely examined sputum eosinophil subpopulations as they relate to exacerbations on mepolizumab and suggests that CD62L^int^ and CD62L^hi^ eosinophils may contribute to break-through exacerbations.

**Figure 2:**
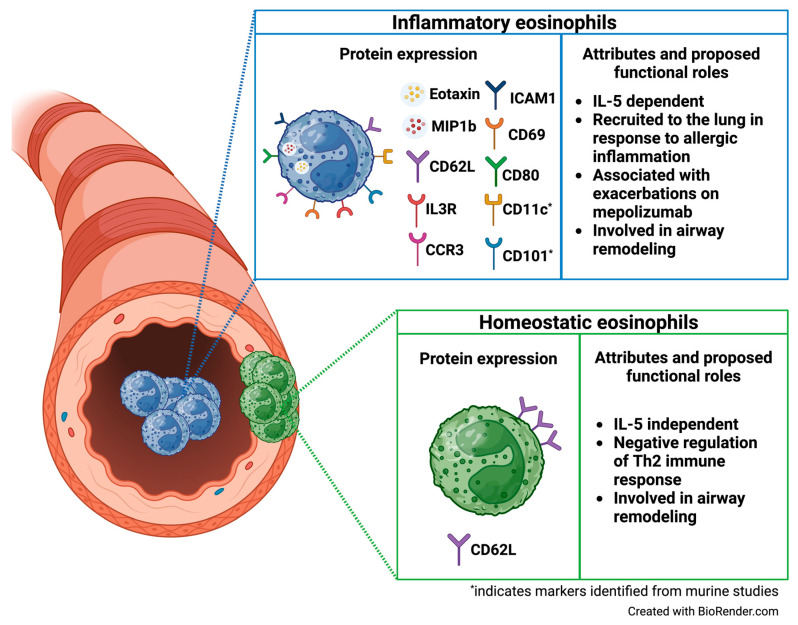
Airway eosinophil subpopulation phenotypes and functions, * indicates markers identified from murine studies. Created with BioRender.com.

These studies provide clear evidence for at least two distinct eosinophil subpopulations both in the lungs (Figure 2) and extrapulmonary tissues; they also highlight the phenotypic and functional diversity between eosinophil subtypes (summarized in Table 1).

## 5. Conclusions and Future Directions 

The studies reviewed here have been instrumental in improving our understanding of eosinophil biology and defining eosinophil subpopulations as they pertain to asthma. Based on their findings, we propose that at least two (and potentially three) distinct eosinophil subpopulations exist in healthy subjects and patients with asthma, chronic rhinosinusitis with nasal polyposis, COPD, RA, and IBD. Regardless of disease or tissue site, there appears to be one predominant “active” or “inflammatory” subpopulation of eosinophils (iEos) that is increased in the setting of infection and inflammation (as seen in the models of IBD and asthma) and one “basal” or “resident” eosinophil subpopulation (“rEos”). Inflammatory eosinophils are characterized by low or intermediate levels of CD62L expression, depending on the tissue context and modality of measurement and are presumed to serve a pro-inflammatory role, whereas resident eosinophils exhibit high levels of CD62L expression and are presumed to perform anti-inflammatory roles important in maintaining tissue homeostasis. Despite these important insights into eosinophil heterogeneity, many questions remain. Do these represent distinct, terminally differentiated eosinophil subtypes, or do they exist on a continuum of stages of maturation? What genetic and environmental factors determine the fate of an eosinophil, beyond the canonical activating cytokine IL-5? Are inflammatory eosinophils responsible for the pathogenesis of eosinophil-associated diseases, and if so, can they be preferentially targeted for therapeutic development? Future studies examining these questions are essential to improving our understanding of eosinophil roles in health and disease and to guiding precision treatments for patients with asthma and other type 2 inflammatory diseases.

## Figures and Tables

**Table 1:** Summary of eosinophil subpopulation studies reviewed.

Study	Eosinophil Subtypes Identified (Method; Sample Type)	Main Findings and Conclusions
Winqvist et al. *Immunology, 1982* Prin et al. *Int Arch Allergy Appl Immunol, 1983* Fukuda et al. *Am Rev Respir Dis, 1985* Kuo et al. *Eur Respir J, 1994*	“Normodense” and “Hypodense” eosinophils (density-gradient centrifugation; human blood)	“Hypodense” eosinophils exhibit an activated phenotype, with decreased eosinophil cationic protein (ECP) content, increased oxygen consumption, increased expression of Fc-IgG and complement receptors, and higher capacity to induce cytotoxicity compared to “normodense” eosinophils [21,22]. “Hypodense” eosinophils are increased in the blood in patients with asthma and correlates with asthma severity and airway hyper-responsiveness [23,24].
Abdala Valencia et al. *Allergy, 2016*	Interstitial eosinophils and airway eosinophils (multicolor flow cytometry; digested lung tissue and BAL specimens from BALBc/J mice)	Airway eosinophils exhibit a Siglec-F^high^CD11c^low^ phenotype compared to Siglec-F^med^CD11c^-^ interstitial eosinophils, indicating that airway eosinophils are phenotypically distinct and that CD11c plays an important role in integrin-mediated trans-epithelial migration of eosinophils to the airways during allergic inflammation [25].
Mesnil et al. *J Clin Invest, 2016*	“rEos” (resident eosinophils) and “iEos” (inflammatory eosinophils) (flow cytometry and FACS sorting; digested lung tissue C57BL/6 mice)	Murine rEos are CD62L^+^ CD101^−^, IL-5-independent eosinophils that reside in the lung parenchyma and exhibit high expression of genes involved in negative regulation of the Th2 immune response. In contrast, iEos are CD62L^−^CD101^+^, IL-5 dependent inflammatory eosinophils that are recruited to the peribronchial areas in response to allergen challenge and exhibit a pro-inflammatory transcriptomic signature. Limited human data indicates variable CD62L expression and low IL3R expression among rEos, whereas iEos express low levels of CD62L and high levels of IL3R [26].
Andreev et al. *Ann Rheum Dis, 2021*	Synovial regulatory eosinophils (“rEos”) and inflammatory lung eosinophils “iEos” (flow cytometry and FACS sorting; digested lung and ankle tissue from WT, ∆dblGATA, and IL-5tf/4Get mice on BALB/cJRj background)	Synovial regulatory eosinophils (“rEos”) accumulate in the synovium in response to allergic asthma and are associated with resolution of arthritis and preservation of joint structure in an IL-5 dependent manner. In a case series of eight patients with rheumatoid arthritis in remission and eosinophilic asthma, six experienced a flare of their RA after initiation of mepolizumab therapy, indicating an immunomodulatory and potentially regenerative role for synovial rEos [27].
Dolitzky et al. *Front Immunol, 2021*	“Type 1-activated” versus “Type 2-activated” eosinophils (peritoneal cavity, IC57BL/6 NJ.1638 *Il5^Tg^* mice)	Type 2 stimulation with IL-4 leads to a distinct transcriptomic signature including upregulation of CD101 and CD69, whereas type 1 stimulation with *E. Coli* and/or IFNɣ led to increased expression of several pro-inflammatory cytokines distinct from that seen with type 2 stimulation, including IL6, IL1b, and TNFα, IL-13, and IL-9 [30].
Gurtner et al. *Nature, 2023*	A-Eos (active eosinophils) and B-Eos (basal eosinophils) (flow cytometry and MACS sorting; digested lung, stomach, colon, and small intestine from C57BL/6J mice)	Active eosinophils (A-Eos) and basal eosinophils (B-Eos) demonstrate tissue-specific localization in the GI tract and distinct transcriptomic signatures, with B-Eos expressing effector molecules involved in tissue morphogenesis and remodeling and A-Eos expressing co-stimulatory molecules involved in immune modulation. A-Eos express high levels of PD-L1 and CD80 and exhibit higher secretory activity, granularity, and activation relative to B-Eos. In gastrointestinal inflammation, A-Eos are increased compared to B-Eos and demonstrate upregulation of genes involved in granulogenesis, antimicrobial activity, immune modulation, IFNɣ signaling, and MHC-1-restricted antigen processing and presentation. A-Eos and B-Eos subpopulations exist on a differentiation continuum, with A-Eos representing the predicted terminal state for all eosinophil subsets. IL-33 is an important factor in inducing maturation of eosinophils to A-Eos [36].
Januskevicius et al. *Cells, 2020* Jurkeviciute et al. *J Pers Med, 2021*	CD62L^+^ rEos and CD62L^−^ iEos (MACS, peripheral blood from patients with asthma and healthy controls)	CD62L^+^ rEos and CD62L^−^ iEos are present in the blood of patients with severe non-eosinophilic asthma (SNEA), allergic asthma (AA), and healthy subjects (HS). While HS exhibit roughly equal proportions of blood rEos and iEos, rEos predominate in the blood of SNEA patients. iEos predominate in the blood of AA patients, but this is attenuated following allergen challenge, suggesting migration of iEos out of the bloodstream and into the tissue in response to allergen challenge. rEos demonstrate increased adhesive properties compared to iEos, supporting their role as resident cells within tissue [37]. Eosinophil subtype integrin expression analysis demonstrates that rEos exhibit higher expression of β1 integrin compared to iEos, which exhibited higher expression of the ɑMβ2 integrin subunits. In co-culture, both eosinophil subtypes significantly increase ASM proliferation, indicating that both may play a role in airway remodeling [40].
Cabrera Lopez et al. *Am J Respir Crit Care Med, 2023*	Siglec-8^+^CD62L^+^IL3R^lo^ rEos and Siglec-8+ CD62L^lo^ IL3R^hi^ iEos, (flow cytometry, peripheral blood from patients with asthma, COPD, smokers without COPD, and healthy volunteers).	Siglec-8^+^CD62L^+^IL3R^lo^ rEos and Siglec-8+ CD62L^lo^ IL3R^hi^ iEos are present in patients with asthma, COPD, smokers without COPD, and healthy volunteers. Circulating iEos are significantly higher in patients with asthma compared to the other groups, regardless of eosinophil count and exhibit increased expression of IL-5Rɑ compared to healthy subjects, smokers without COPD, and patients with COPD [41].
Matucci et al. *Clin Exp Allergy, 2023* Vultaggio et al. *Allergy, 2023*	CD62L^bright^ rEos and CD62L^low^ iEos (FACS, peripheral blood and nasal polyp tissue from patients with severe eosinophilic asthma with or without chronic rhinosinusitis with nasal polyposis)	CD62L^low^ iEos are increased in the blood of patients with severe eosinophilic asthma compared to healthy donors and are highly concentrated in nasal polyp tissue from patients with concomitant chronic rhinosinusitis with nasal polyposis. CD62L^low^ iEos from the blood demonstrate increased expression of CCR3 and CD69, and decreased expression of IL-5Rɑ, CRTH2, CD86, and CD28 when compared to CD62L^bright^ rEos, supporting the notion that iEos reflect an activated and functional subset of eosinophils. The proportion of CD62L^low^ iEos correlate with asthma and nasal polyp scores and number of asthma exacerbations, implicating iEos as a potential biomarker for risk of exacerbations and poor asthma control [42]. Stimulation with IL-5 significantly increases the percentage of iEos in the blood, and exposure to IL-5 blockade with mepolizumab, and an anti-IL-5R antibody abrogates this expansion of iEos. In patients with severe eosinophilic asthma, treatment with mepolizumab results in a significant and sustained reduction in the proportion of CD62L^low^ eosinophils in the blood. This reduction in iEos following mepolizumab directly correlates with improvement in post-treatment asthma control scores, indicating that mepolizumab may improve asthma control through a direct effect on reducing circulating iEos [43].
Wilson et al. *J Allergy Clin Immunol, 2024*	CD62L^hi^ eosinophils, CD62L^int^ eosinophils, and CD62L^lo^ eosinophils (mass cytometry (CyTOF), sputum from children with exacerbation-prone eosinophilic asthma enrolled in the MUPPITS2 clinical trial and treated with mepolizumab or placebo)	Three distinct airway eosinophil subpopulations exist and express low, intermediate, and high levels of CD62L (“CD62L^lo^”, “CD62L^int^”, and “CD62L^hi^” eosinophils). CD62L^int^ eosinophils express increased levels of several chemokine receptors and ligands involved in eosinophil activation and mobilization (including CD69, CD80, ICAM1, MIP1β, Eotaxin, and CCR3) compared to the other airway eosinophil subpopulations. Among children treated with mepolizumab, CD62L^int^ and CD62L^hi^ eosinophils are increased in those who experience exacerbations on treatment compared to exacerbation-free children, indicating that these eosinophil subpopulations may play a role in break-through exacerbations on mepolizumab [45].
